# The Cell Cycle Time of CD8^+^ T Cells Responding *In Vivo* Is Controlled by the Type of Antigenic Stimulus

**DOI:** 10.1371/journal.pone.0015423

**Published:** 2010-11-08

**Authors:** Heesik Yoon, Taeg S. Kim, Thomas J. Braciale

**Affiliations:** 1 The Carter Immunology Center, University of Virginia, Charlottesville, Virginia, United States of America; 2 Department of Microbiology, University of Virginia, Charlottesville, Virginia, United States of America; 3 Department of Pathology, University of Virginia, Charlottesville, Virginia, United States of America; Université de Toulouse, France

## Abstract

A hallmark of cells comprising the mammalian adaptive immune system is the requirement for these rare naïve T (and B) lymphocytes directed to a specific microorganism to undergo proliferative expansion upon first encounter with this antigen. In the case of naïve CD8^+^ T cells the ability of these rare quiescent lymphocytes to rapidly activate and expand into effector T cells in numbers sufficient to control viral and certain bacterial infections can be essential for survival. In this report we examined the activation, cell cycle time and initial proliferative response of naïve murine CD8^+^ T cells responding *in vivo* to Influenza and Vaccinia virus infection or vaccination with viral antigens. Remarkably, we observed that CD8^+^ T cells could divide and proliferate with an initial cell division time of as short as 2 hours. The initial cell cycle time of responding CD8^+^ T cells is not fixed but is controlled by the antigenic stimulus provided by the APC *in vivo*. Initial cell cycle time influences the rate of T cell expansion and the numbers of effector T cells subsequently accumulating at the site of infection. The T cell cycle time varies with duration of the G_1_ phase of the cell cycle. The duration of G_1_ is inversely correlated with the phosphorylation state of the retinoblastoma (Rb) protein in the responding T cells. The implication of these findings for the development of adaptive immune responses and the regulation of cell cycle in higher eukaryotic cells is discussed.

## Introduction

Naive CD8*^+^* T cells typically respond to a specific antigenic stimulus through a stepwise sequence of events starting with activation and associated blast transformation of the resting lymphocytes [1]–[3]. This is followed by multiple rounds of proliferation of the responding T cells and, if the appropriate signals are delivered, to differentiation of the cells into activated effector T cells [4], [5]. The encounter of the naïve CD8*^+^* T cells with foreign antigen and the subsequent proliferative expansion leading to effector cells generation occurs primarily within organized secondary lymphoid tissues such as lymph nodes and spleen [6], [7].

The interaction of naïve CD8*^+^* T cell with antigen bearing antigen presenting cells (APC) results in a complex series of signaling events mediated by both soluble and cell bound ligands [1], [8]–[10]. The APC likely plays a dominant role in controlling the subsequent fate of the responding T cells. The nature and the activation/maturation state of the APC as well as the duration of T cell-APC contact affect T cell proliferative expansion and the clonal burst size of the CD8*^+^* effector cells generated [1], [11]. Accessory interactions within the architectural confines of the secondary lymphoid structure in which the T cell-APC encounter occurs also undoubtedly affect T cell activation, proliferation and the overall quality of the subsequent CD8*^+^* T cell response [12].

In lymphocytes as with all other eukaryotic cell types, the program for cell division is controlled at multiple regulatory points throughout the cell cycle [13], [14]. One of the more prominent and critical regulatory points controlling the onset and tempo of cell division is the transition from the G_1_ into the S phase of the cell cycle and the onset of DNA synthesis [15]–[18]. As a result the duration of time in which a cell is in the G_0_/G_1_ phase of the cell cycle will greatly affect the speed of cell division. Studies of cell division times in lymphocytes have primarily been carried out *in vitro* by stimulating heterogeneous populations of T cells with mitogens or crosslinking anti-receptor antibodies, or in some instances TCR transgenic (tg) T cells using a specific antigenic stimulus [8], [19]. In general these studies demonstrate initial cell cycle times for activated lymphocytes (following blast transformation) of 6–8 hrs. More recently, the doubling time of naïve CD8*^+^* T cell precursors *in vivo* to an infectious antigenic stimulus has been estimated to be as short as 4 hrs [20], [21].

Available evidence suggests that, within a given individual, the number of naïve CD8*^+^* T cells directed to a given epitope ranges from several hundred to ∼1,000 cells distributed throughout the lymphoid compartments [22]–[24]. Therefore, relatively few naive precursors are likely to be directed to a given invading pathogenic microorganism. Consequently, there should be strong selective pressure to mobilize the few naïve precursors available and trigger cell division and proliferative expansion of the precursors into effector cells as rapidly as possible. It would be anticipated then that the cell cycle time of T cells responding *in vivo* to any antigenic stimulus may be extremely short. However, cell cycle times for T cells responded *in vivo* have not been extensively analyzed [25], [26], and it is not known whether the division time *in vivo* of antigen specific T cells is uniformly fixed or varies with antigenic stimulus and the duration of cell proliferation.

In this report, we have examined the early proliferative response and cell cycle time of naïve CD8*^+^* T cells responding *in vivo* within secondary lymphoid organs, e.g., the draining lymph nodes (DLN) and spleen, to several infectious and non infectious stimuli. We find that with a potent antigenic stimulus such as infection with Type A influenza virus, T cells initiate proliferation with cell cycle times of as short as 2 hrs. Initial cell cycle times are not fixed, but vary with the nature of the antigenic stimulus and control of cell cycle time is dictated by the duration of the G_1_ phase of the cell cycle. Rapid initial cell proliferation is associated with accelerated T cell expansion and as a consequence increased accumulation of effector cells at the site of initial antigen deposition/infection in the lungs. Responding CD8^+^ T cells with fast initial cell cycle time differed in transcriptome profile from the responding cells with slow initial cell cycle time. This profile difference was reflected in the level of expression of genes encoding positive and negative regulators of G_1_/S phase transition. Furthermore, the cell division time and the duration of G_1_ was correlated with the level of phosphorylated Rb (retinoblastoma) protein within the responding cells. The implications of these findings are discussed.

## Results

### Responding CD8*^+^* T cells rapidly proliferate in the draining lymph nodes

We have previously examined the CD8*^+^* T cell response to intra nasal (i.n.) influenza infection by adoptive transfer of CFSE labeled naïve influenza HA specific TCR-transgenic Clone 4 (CL-4) CD8*^+^* T cells into normal recipients [20], [21]. We observed that following the onset of proliferation of the naïve T cells in the DLN (at approximately day 3 post infection [p.i.]), the T cells had undergone approximately 6–7 divisions over the ensuing 24 hrs (4 day p.i.) suggesting extremely rapid cell division by the responding T cells during this 24 hr interval.

**Figure 1:**
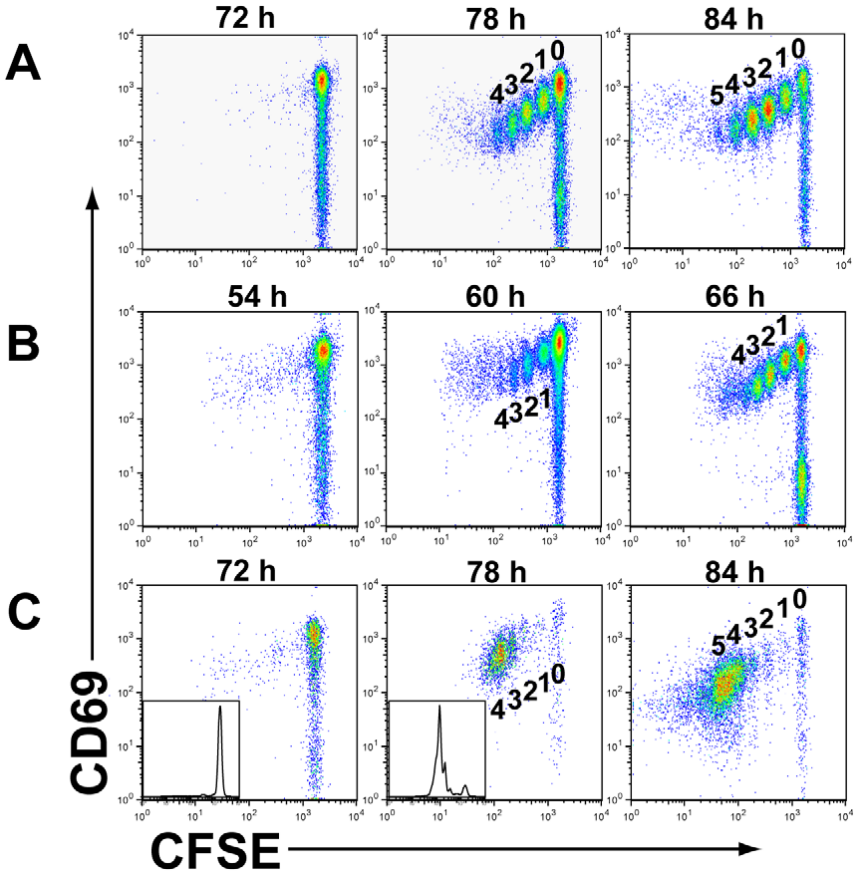
Tempo of early CD8*^+^* T cell division in the DLN following influenza infection. A. *In vivo* division time of TCR tg CL-4 T cells responding in the DLN to influenza A/PR8 at the indicated times p.i. as determined by CFSE dye dilution. Results are representative of 6 independent experiments using 2–3 mice/exp. with mean division numbers between 72 hrs and 78 hrs pi of 3.19 ± 0.17 divisions over the 6 hr time frame. Percentages of cells at each division (#1 through 4) at 78 hr time point are 13.1% (division #1), 11.7% (#2), 8.5% (#3), and 3.5% (#4) of total CL-4 CD8^+^ T cells per DLN, respectively. B. *In vivo* division time of TCR tg Demi-4 T cells in the DLN responding to influenza A/JAPAN and analyzed as described above at the indicated times p.i. Results are representative of 3 individual mice analyzed in 2 experiments with a mean of 3.0 ± 0.1 divisions between 54 hrs and 60 hrs p.i. C. *In vivo* division time of a single cohort of CFSE labeled TCR tg CL-4 T cells responding in the DLN to infection after administration of anti CD62L mAb at 24 hrs p.i. Results are representative of 6 independent experiments with pooled DLN from 4 mice per experiment. Inserts show CFSE intensity (X-axis) and cell numbers as % of maximum (Y-axis) at the indicated time-points.

To further evaluate the *in vivo* cell cycle time of the responding CD8*^+^* T cells, we used the same strategy to monitor cell division in the DLN by CFSE dilution at 72, 78 and 84 hrs p.i. with a 0.1 LD_50_ dose of influenza A/PR/8 virus. We found that between 72 hrs p.i. (when the naive CD8*^+^* T cells in the DLN had activated, but not as yet initiated their first division) and 78 hrs p.i., the responding CD8*^+^* T cells had undergone up to 4 doublings (as determined by CFSE dilution) within this 6 hr time window (Figure 1A). These data are representative of 6 independent experiments with a mean number of 3.19 ± 0.17 cell divisions between 72 and 78 hrs p.i. by the responding T cells. Therefore the responding T cells had divided with an apparent cell cycle time of ∼2 hrs. Of note, the rate of cell division also appeared to begin to slow after 4–5 cell doublings (see below).

We considered alternative (potentially trivial) explanations for this unexpected result including an inability to liberate proliferating T cells by mechanical disruption of the lymph nodes selectively at the 72 hr time-point or recruitment of proliferating cells from other sites into the DLN during the 6 hr interval between 72 and 78 hrs. There was no difference in the distribution of the CFSE labeled cells when the transgenic CD8*^+^* T cells were liberated from the DLN by the mechanical disruption or enzymatic digestion (Figure S1A). Furthermore, in keeping with our published results [20] we were unable to detect proliferating T cells in sites other than the DLN up to 96 hrs (day 4) p.i. (Figure S1B). Also, we observed the same rapid cell cycle time, when a10-fold lower inocula of CL-4 T cells were used for transfer (Figure S1C). We noted at the 72 hr time point p.i. (left panel Figure 1A) a small number of “cells” (events) which lay within the CD8^+^ T cell flow cytometry gate and which already had diminished CSFE intensity. These events probably represent cells that had already become activated by 72 hrs and had undergone 1–3 divisions. Since these cells constitute less than 2% of the CFSE-labeled CD8^+^ T cells in the DLN, however, additional division of these cells between 72 and 78 hrs would only contribute very marginally to the accumulation of rapidly dividing cells observed at the 78 hr time point. Furthermore, additional division of the cells would be associated with even further CFSE dilution which does not reflect the pattern of dye dilution (cell division) observed at 78 hrs.

This finding of extremely rapid cell division and short cell cycle time was not a unique property of the TCR tg CL-4 T cells or a unique feature of the response to the influenza A/PR/8 virus used for infection. We observed comparable rapid divisions by responding CD8*^+^* T cells obtained from another independent influenza-specific TCR tg CD8*^+^* T cell mouse line Demi-4. Like the CL-4 T cells the Demi-4 T cells exhibit a cell cycle time of ∼2 hrs (Figure 1B). These CD8*^+^* T cells recognize a hemagglutinnin epitope (JHA_210–219_) from the A/JAPAN virus strain. After i.n. infection with the A/JAPAN virus the onset of CD8*^+^* T cell proliferation in the DLN occurs between 54 hrs and 60 hrs p.i. This reflects the fact that this influenza virus strain reaches high steady state viral titers in the mouse respiratory tract more rapidly than the A/PR/8 virus and therefore triggers respiratory dendritic cell (RDC) migration to the DLN to initiates T cell activation more rapidly [21].

We have previously reported that naïve virus-specific CD8*^+^* T cells responding to i.n. influenza virus infection enter the DLN from the circulation, activate, proliferate and exit from DLN sequentially [21]. Furthermore, by blocking L-selectin on the circulating naïve CD8*^+^* T cells at a specific time p.i., we could restrict the proliferative response to a single cohort of naïve CD8*^+^* T cells which had entered the DLN prior to L-selectin blockade [21]. If the responding CD8*^+^* T cells in the DLN proliferate with the cell cycle time less than or equal to 2 hrs, then a single cohort of responding CD8*^+^* T cells entering the DLN should divide (i.e., dilute CFSE) at a rate consistent with this rapid cell cycle time. To test this possibility, we transferred CFSE labeled naïve CL-4 T cells into recipients and 24 hrs p.i. when viral antigen bearing respiratory dendritic cells (RDC) had entered the DLN, we blocked additional CL-4 T cell migration into DLN *in vivo* by administration of anti L-selectin antibody, Mel-14. The proliferative response of the initial cohort of responding T cells in the DLN was analyzed at 72, 78, and 84 hrs p.i. As Figure 1C demonstrates, the synchronous cohort of proliferating CD8*^+^* T cells underwent 3–4 divisions in the 6 hr time interval between 72 and 78 hrs p.i. This finding, along with the above results, suggest that the CFSE dilution pattern observed among the proliferating T cells in the DLN reflects this extremely short cell cycle time. These results also make less likely the possibility that a small fraction of CL-4 T cells already in active cell division at 72 hrs p.i. in the draining lymph nodes account for the apparent rapid (∼2 hr) cycle time observed.

### Cell division, DNA content and DNA synthesis

Cells undergoing rapid division with a short cell cycle time characteristically demonstrate only a limited fraction of the cells in the G_1_ phase of the cell cycle — that is, a large percentage of the cells are carrying out DNA synthesis and therefore in S/G_2_ phase of the cell cycle [28], [29]. If the activated CD8*^+^* T cells in the DLN proliferated with a cell cycle time of ∼2 hrs, then only a small fraction of cells should be in G_1_ (i.e., display 2N DNA content). To evaluate this, we analyzed the DNA content of responding CL-4 T cells in the DLN at 78 hrs p.i. gating on proliferating cells (through division #5) as well as activated undivided cells (division #0) based on CFSE intensity. Resting naïve CL-4 T cells in the non-draining lymph nodes (NDLN) served as a control for cells in G_0_/G_1_ with 2N DNA content.

**Figure 2:**
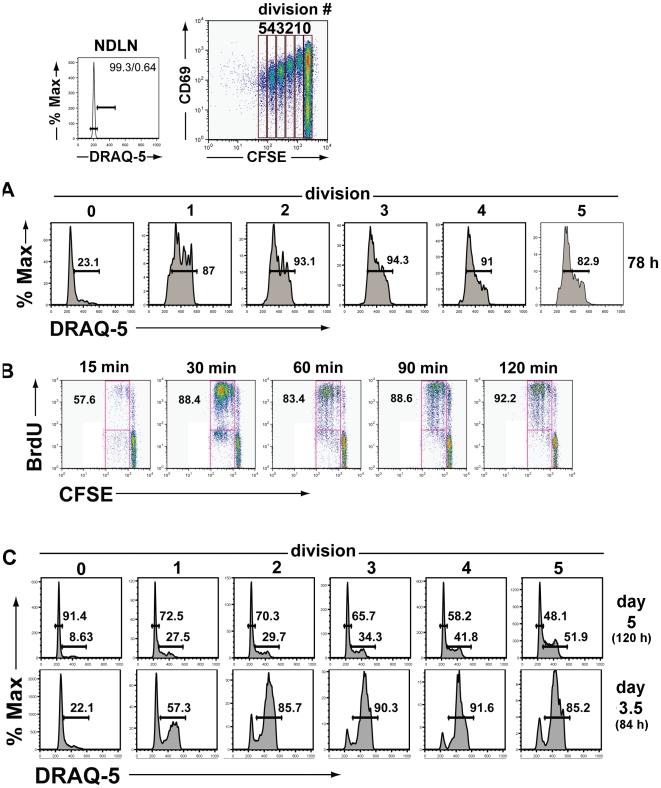
DNA content and DNA synthesis by responding CD8*^+^* T cells in the DLN. A. Adoptively transferred CFSE labeled CL-4 T cells were harvested from the DLN of infected mice at 78 hrs p.i. Responding cells were analyzed by flow cytometry and gated as to division number (0 through 5) based on CFSE dye intensity. DNA content was assessed by uptake of the cell permeable fluorescent dye DRAQ-5, a highly cell permeable DNA-interactive agent. Upper right panel shows the gating strategy used to identify and analyze individual dividing cell populations. Upper left panel shows DNA content of naive CL-4 T cells (in G_0_/G_1_) isolated from the NDLN of infected mice. Numbers shown are the percentages of cells in S*+*G2/M. Results are representative of the compiled experiments described in Table S1. B. At the indicated times following the i.v. administration of the thymidine analog BrdU at 78 hrs p.i., the DLN of infected recipients of CFSE dye labeled CL-4 T cells were harvested and the T cells were analyzed for CFSE intensity (cell division) and BrdU uptake (DNA synthesis activity). Data are representative of 4 independent experiments with 2–3 mice/exp. Values represent the percentage of BrdU*^+^* cells among the dividing cell population. C, Results as in Figure 2A except that the DLN were harvested at days 3.5 and 5 p.i., respectively. This comparative analysis is representative of 2 independent experiments with 3 mice/exp. Numbers shown are the percentages of cells in S*+*G_2_/M (> 2N DNA content) and the percentages of cells in G_1_ (2N DNA content) for day 5 p.i. cells and the percentages of cells in S*+*G_2_/M (> 2N DNA content) for day 3.5 p.i. cells.

As Figure 2A demonstrates, proliferating T cells within the gates encompassing divisions 1 through 5 could have up to 94% of the cells with >2N DNA content. This finding suggests that these rapidly dividing cells progress through the cell cycle with minimal time in G_1_. These results are in agreement with our CFSE intensity data suggesting a rapid cell division time for these CD8*^+^* T cells. While transferred CD8*^+^* T cells in the NDLN retained 2N DNA content (and were presumably in G_0_), the undivided but activated (CD69*^+^*) tg T cells in the DLN consisted of cell populations with either 2N DNA content or cells with >2N DNA. The latter population likely represents responding T cells which have entered S phase and initiated DNA synthesis prior to the first cell division. Summaries of the percentage of cells in S*+*G2/M, i.e., with > 2N DNA content among the responding CD8*^+^* T cells in the DLN at 78 hrs p.i. are included for replicate experiments in Table S1.

The presence of such a large fraction of dividing cells in the DLN with > 2N DNA suggests that the majority of cells are actively synthesizing DNA. To directly evaluate this, we administered the thymidine analog BrdU i.v. into CL-4 T cell recipients at 78 hrs p.i., and excised the DLN at intervals between 15 minutes and 2 hrs after analog administration. As Figure 2B demonstrates BrdU had been taken up by approximately 50% of the dividing cells by as early as 15 minutes after analog administration and maximum BrdU incorporation into DNA (i.e., 80%–90% BrdU*^+^*) in the dividing CL-4 T cells was observed between 15 and 30 minutes after analog administration. It is noteworthy that a fraction of the as yet undivided but activated CD8*^+^* T cells in the DLN also had taken up BrdU — a finding consistent with our observation on the content of DNA in this responding T cell population. As expected, no BrdU uptake was observed in CL-4 T cells isolated from the NDLN (Figure S2).

### Cell cycle times *in vivo* are not fixed

Our results suggested that upon entry into the DLN and activation, virus-specific CD8*^+^* T cells can undergo extremely rapid initial division with very short cell cycle times. However, as these T cells continued to proliferate and accumulate in the DLN between day 3 and 5 p.i., we found that the rate of cell division appeared to decrease (and the cell cycle time increased). For CD8*^+^* T cells which entered the DLN early after infection and initiated proliferation between 72 hrs (day 3) and 84 hrs (day 3.5) p.i., this increase in the cell cycle time (and slower division rate) became evident after the cells had undergone 5 or more divisions (Figures. 1 and 2A). For virus-specific T cells which entered the DLN at later times p.i. (e.g., day 5 p.i.) and underwent activation and proliferation in the DLN, an increased cell cycle time (and slower division rate) was evident for all activated dividing cells independent of number of divisions which the cells had undergone. As Figure 2C demonstrates, 50–70% of the proliferating T cells analyzed on day 5 p.i. were in G_1_ (i.e., had 2N DNA content) while less than 15% of the proliferating T cells at day 3.5 p.i. were in the G_1_ phase of the cell cycle.

**Figure 3:**
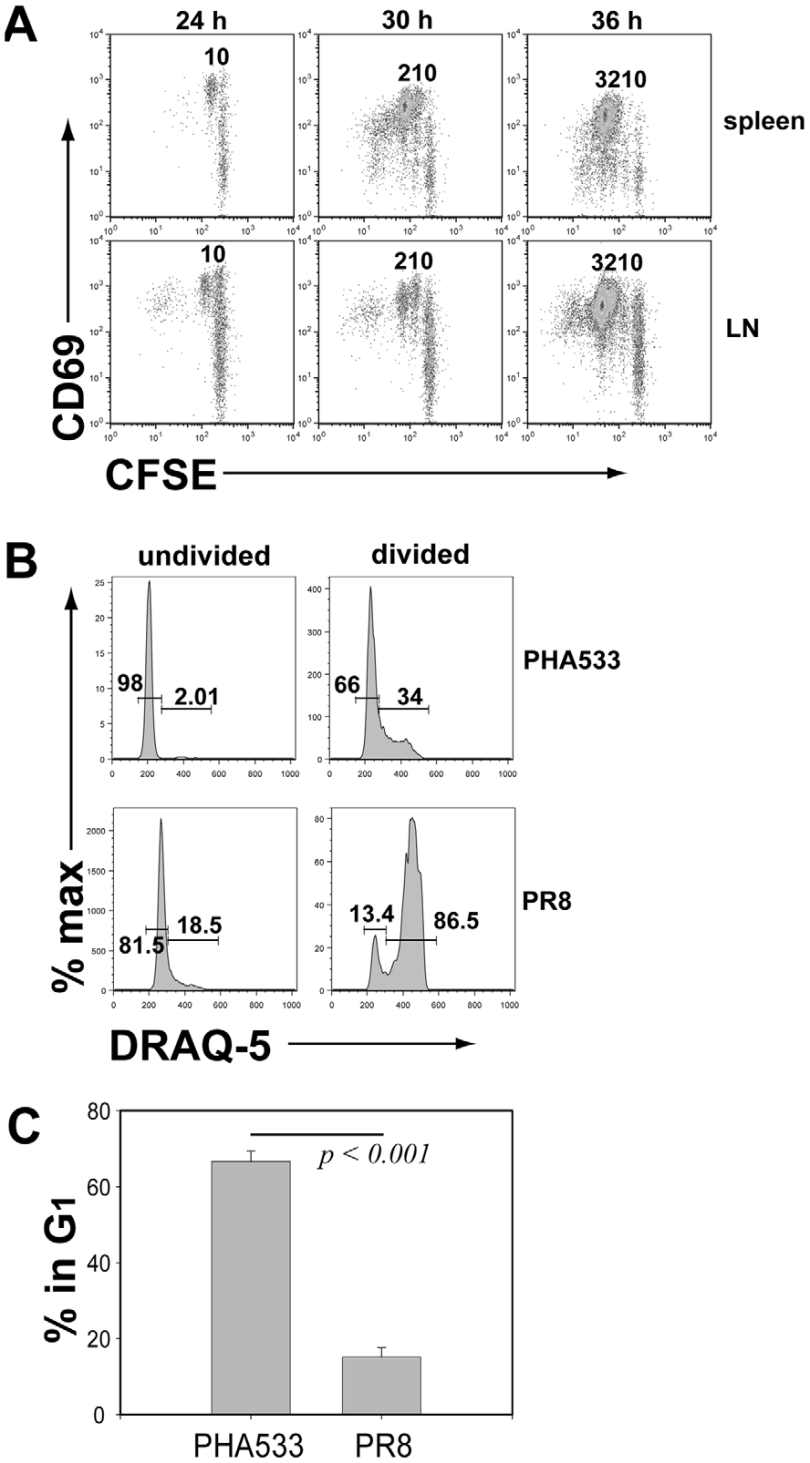
Cell division rate, DNA content and cell cycle phase. A. Pooled lymph nodes and spleen were harvested at the indicated times from recipients of dye labeled CL-4 T cells after i.v. administration of 100–250 µl of 1 µM soln. of synthetic PHA_533–541_ peptide and the responding T cells analyzed as described in Figure 1. Results are representative of 10 independent experiments (n = 1–2 animals/experiment). Mean values for the division numbers from pooled lymph nodes and spleens are 0.66 ± 0.08 divisions from 0 hrs to 24 hrs post peptide administration, 1.28 ± 0.03 divisions for the 6 hr interval from 24 hrs to 30 hrs, and 0.99 ± 0.01 divisions between 30 hrs and 36 hrs after peptide administration. B. DNA content of CL-4 TCR tg T cells isolated either from the lymph nodes of T cell recipient mice 36 hrs after i.v. administration of synthetic peptide (PHA_533–541_) or from the DLN of mice infected with influenza A/PR/8 and harvested 3.5 days (84 hr) p.i. For each group the cells are gated as divided or undivided cells and the fraction of cells with 2N or > 2N DNA content determined. Data are representative of 4 independent experiments with 3–4 mice/exp. Numbers shown are the percentages of cells in G_1_ or S*+*G_2_/M (shown are values for LN cells only). C. Percentage of dividing CL-4 T cells in the G1 phase of the cell cycle for peptide immunized or infected cell donors analyzed 3.5 days p.i. as described in Figure 3B. Values are the mean percentages from peptide treated animals (n = 13) or infected animals (n = 9) pooled from 4 independent experiments.

These results raised the possibility that the cell cycle time of the CD8*^+^* T cells responding to antigen may not be uniform *in vivo.* To evaluate whether in addition to the number of accumulated divisions, the nature of the antigenic stimulus influenced the cell cycle time of the T cells, we immunized CL-4 T cell recipients i.v. with the synthetic peptide corresponding to the target influenza hemagglutinin epitope (PHA_533–541_) recognized by CL-4 cells and examined T cell proliferation *in vivo* (in spleen and lymph nodes) during the first 36 hrs after immunization. As Figure 3A demonstrates by 24 hrs after immunization, the naïve T cells had activated (i.e., upregulated CD69) and initiated one round of cell division in lymph nodes and spleen in response to peptide. However, over the next 12 hrs the proliferating cells underwent only two additional divisions indicating an initial cell cycle time of approximately 6 hrs. In contrast to virus infection where virus replication, uptake of viral antigen by RDC and RDC migration to the DLN precedes the sequential process of encounter of the T cell with the antigen bearing dendritic cell [20], [21], [30], because of the immediate accessibility of the peptide antigen to secondary lymphoid organs, the naïve T cells simultaneously responded both in lymph nodes and spleen.

The longer cell cycle time of T cells responding to peptide stimulation was also evident from the analysis of the fraction of dividing cells in G_1_ (Figure 3 B and C). Approximately 60–70% of the proliferating T cells, stimulated *in vivo* by peptide were in the G_1_ phase of the cell cycle while by comparison, following influenza infection, <20% of the early proliferating (at day 3.5) CL-4 T cells in the DLN were in G_1_ (Figure 3 B and C). Of note, the division rate of CL-4 cells responding to peptide immunization *in vivo* directly paralleled the cell cycle time of naïve CL-4 T cells responding *in vitro* to peptide stimulation or virus infected cells i.e., ∼6 hrs (data not shown).

**Figure 4:**
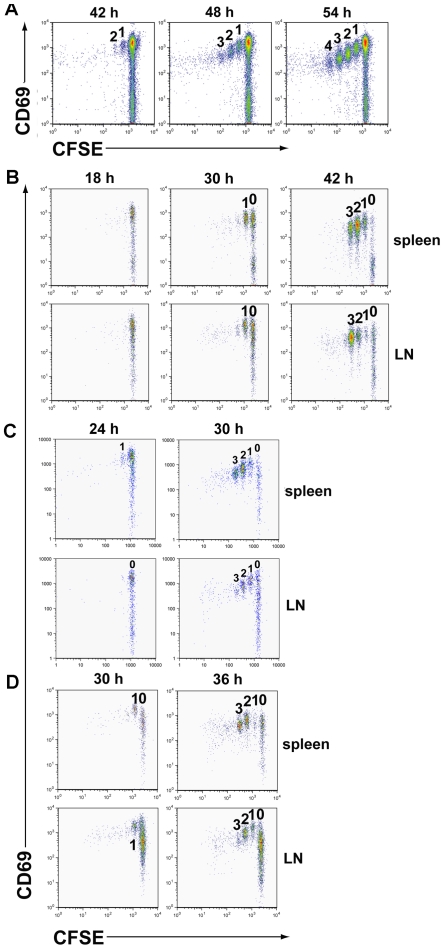
Effect of antigenic stimulus on the initial division rate of responding T cells. A. Recipients of CFSE labeled CL-4 T cells were infected i.n. with 10^7^ pfu of recombinant vaccinia virus expressing the HA of A/PR/8. At the indicated times post infection the DLN were harvested and CL-4 T cell proliferation assessed. Results are representative of 4 independent experiments (n = 2–3 mice per experiment) with mean number of divisions of 1.55 ± 0.25 between 42 and 48 hrs p.i. and 1.0 ± 0.2 divisions between 48 hrs and 54 hrs p.i. B. Experimental design as in Figure 3A except that the PHA_533–541_ peptide was delivered i.n. in a cocktail of TLR-3, 7 and 9 ligand agonists adjuvants as described (Materials and Methods). Data are representative of 2 independent experiments using 3 animals/exp. C and D. Influenza A/PR/8 infected splenic DC (C) or infectious virus (D) was delivered i.v. to recipients of CFSE labeled CL-4 T cells. At the indicated times post administration of DC or virus, lymph nodes and spleen were harvested and the proliferative response of the T cells determined. Results are representative of 2 independent experiments and a total of 6 animals. The mean number of CL-4 T cell divisions was 1.97 ± 0.2 between 30 hrs and 36 hrs for i.v. infectious virus administration.

Since influenza virus infects the respiratory tract and the adoptive immune T cell responses are initiated in the draining mediastinal lymph nodes [20], [30], it was of interest to determine if rapid cell cycle time was a common feature of the CD8*^+^* T cell response in the DLN to respiratory tract infection with another virus. To evaluate this possibility, recipients of CFSE labeled CL-4 cells were infected with a recombinant vaccinia virus expressing the influenza A/PR/8 hemaglutinnin (VV-HA) and the tempo of CL-4 proliferation in the DLN analyzed. Induction of a potent CD8*^+^* T cell response to this recombinant vaccinia virus required a high i.n. inoculum dose (i.e., 10^7^ pfu of virus). Infection with this high virus inoculum produced rapid mobilization and migration of RDC to the DLN. As a result, the onset of detectable CL-4 T cell proliferation in the DLN was somewhat more variable, but typically occurred at least a full day earlier (i.e., at 42 hrs p.i.) than for influenza A/PR/8 infection (i.e., 78 hrs p.i.). As Figure 4A demonstrates, in spite of the earlier onset of CD8*^+^* T cell response to vaccinia infection, the initial proliferation of the cells in the DLN occurs with a cell cycle time of approximately 5 - 6 hrs. These results suggest that rapid initial T cell proliferation and extremely short cell cycle times was not an intrinsic property of CD8*^+^* T cells responding *in vivo* in the DLN to any infectious antigenic stimulus.

### Cell cycle time varies with the antigenic stimulus

Although both influenza and vaccinia virus replicate in the respiratory tract and stimulate a CD8*^+^* T cell response in the DLN, infection with the attenuated vaccinia virus drives CD8*^+^* T cell proliferation with the slower initial cell cycle time than does influenza infection. In view of these results and the results with peptide immunization, we considered the possibility that the cell cycle time of responding CD8*^+^* T cells might be dictated at least in part by the type of antigenic stimulus.

To evaluate this we determined whether the initial rate of CL-4 T cell division following immunization with the synthetic PHA_533–541_ peptide could be accelerated by administration of the peptide in an adjuvant. We vaccinated recipients of CFSE labeled naïve CL-4 T cells with the PHA_533–541_ peptide adjuvanted with TLR 3, 7, and 9 agonists either individually or as an adjuvant cocktail and compared immunization by the i.v., and i.n. routes. As Figure 4B demonstrates for i.n. inoculation with the peptide/TLR adjuvant cocktail, initial cell division time in response to peptide vaccine was uniformly ∼6 hrs for any combination of adjuvants and either route of inoculation (data not shown).

Similarly, immunization with peptide pulsed splenic dendritic cells either with or without prior LPS induced maturation also did not shorten the 6 hr initial cell cycle time of the T cells responding to synthetic peptide (data not shown). By contrast, i.v. immunization with splenic dendritic cells infected with the A/PR/8 virus, stimulated initial CD8*^+^* T cell division with a cell cycle time of ∼3–4 hrs (Figure 4C) which is faster than peptide pulsed DC but slower than the i.n. influenza infection. Similar results were obtained when mice for immunized with infectious influenza virus by the i.v. route (Figure 4D).

### Effect of the cell cycle time on CD8*^+^* T cell responses

**Figure 5:**
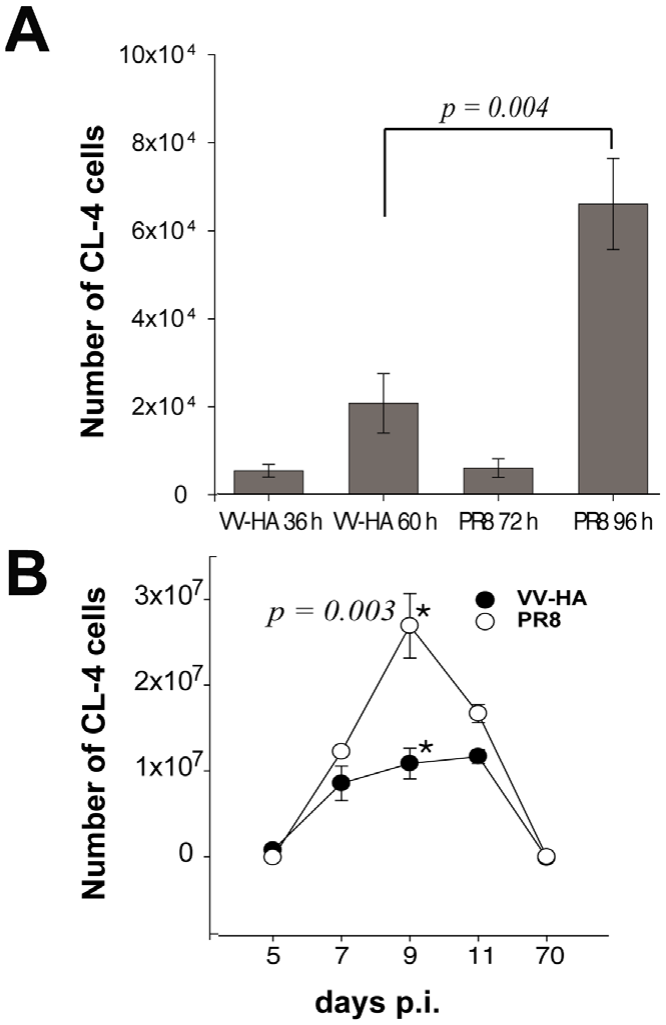
Cell cycle time and T cell expansion. A. CL-4 T cells were transferred into Thy1 congenic naïve recipients. Following i.n. infection with VV-HA or A/PR/8 virus, the DLN were harvested either immediately prior to the onset of T cell proliferation (36 hrs for VV-HA and 72 hrs 4 A/PR8) or 24 hrs thereafter. Total CL-4 T cells in the pooled DLN (2–3 donors per experiment) were enumerated at the two indicated time points. Values are the mean cell numbers from 4 experiments. B. Total CL-4 T cells accumulating in the VV-HA or Influenza infected lungs were enumerated on the indicated days. Values are pooled results from 3 independent experiments using 3–4 mice/exp.

The difference in the initial cell cycle time of CD8*^+^* T cells responding to i.n. influenza and vaccinia infection provided us an opportunity to explore the impact of the rate of cell division and cell cycle time on the pattern of the CD8 T cell response in the DLN and lungs after i.n. virus infection. As a first step we analyzed the accumulation of CL-4 T cells in the DLN during the 24 hr period marking the initial onset of proliferation of the responding T cells in the DLN. For vaccinia infection, this time-frame was from 36 hrs to 60 hrs p.i. and for influenza infection this time was from 72 hrs to 96 hrs p.i. As Figure 5A demonstrates, there was an increase in the number of CL-4 T cells detected in the DLN over the initial 24 hr period in both infected groups. However, the total accumulation of the responding CD8*^+^* T cells was substantially higher in the DLN of influenza infected animals (> 3 fold) than in the DLN of vaccinia infected animals within the first 24 hrs following the onset of T cell division. This result was consistent with our finding of a difference in cell cycle time of the T cells responding to the two viral stimuli.

Activated effector CD8*^+^* T cells begin to emigrate from the DLN to the infected lungs on day 5–6 p.i. and continue to accumulate in the infected respiratory tract over the next 3–4 days [20]. When the absolute number of CL-4 T cells present in the lungs of influenza and vaccinia infected mice were analyzed over time, we found that the responding CL-4 T cells had accumulated to substantially higher numbers by day 9 p.i. in the lungs of influenza infected mice compared to the vaccinia infected animals (Figure 5B). However, we did not detect any difference in the expression of cell surface activation/differentiation markers or in effector cytokine production by the T cells responding to either the influenza or the vaccinia stimulus (data not shown).

### Gene expression profile of T cells undergoing rapid or slow initial cell division

For T cells responding to an antigenic stimulus in vivo, our results suggested that the initial rate of division of the activated cells was not fixed but variable and, in part at least, dictated by the type of the antigenic stimulus. Our data further suggested that in these responding T cells cell division time was linked to the duration of G_1_. We wanted to determine if the CD8^+^ T cells responding with different initial rates of proliferation differed in their profiles of expressed genes particularly the expression of genes controlling the transition of cells from the G_1_ to the S phase of the cell cycle. To do so we carried out a transcriptome profile of T cells undergoing rapid initial cell division, i.e., CL-4 T cells isolated from the DLN of influenza infected mice at 78 hrs p.i., and T cells undergoing slower initial cell division, i.e., CL-4 T cells isolated from the LN of mice immunized with synthetic HA peptide epitope.

**Figure 6:**
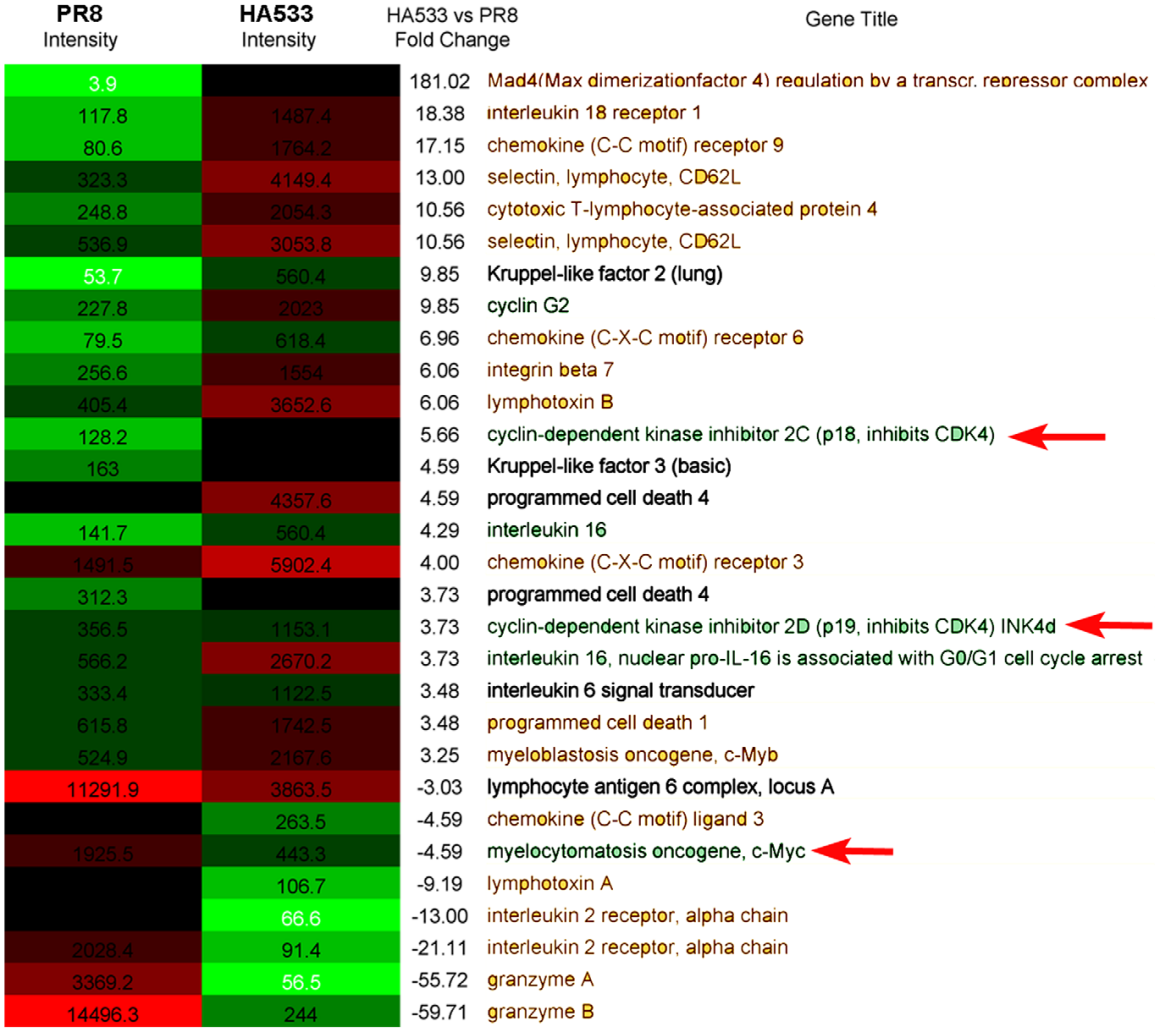
Expression array profile of proliferating T cells. CFSE labeled CL-4 T cells undergoing divisions 1 through 4 were isolated by cell sorting from the DLN of influenza infected mice (at 78 hrs p.i.) and from the LN of peptide immunized mice (at 36 hrs after peptide administration). RNA was extracted from the sorted cells (> 95% purity) and subjected to expression array analysis. Values are signal intensities for selected genes differing by 3 or more fold between the 2 comparison groups. The DLN of 8 mice/group served as the source of pooled CL-4 T cell RNA for analysis.

We found that more than 430 genes were differentially expressed (more than three-fold) in these two early activated T cell populations. Thirty of these genes were associated with either T cell activation/differentiation state or cell cycle regulation and are displayed in Figure 6. The elevated expression of certain genes (e.g. *granzyme A/B*) in the T cells activated through virus infection would suggest that these responding cells might be driven further along the activation/differentiation pathway than peptide stimulated T cells. However, of more interest for us was the differential expression of several genes linked to control of cell cycle at the G_1_/S interface. Specifically, genes encoding two inhibitors of G_1_/S interface cyclin dependent kinases (i.e., *p18INK4c and p19INK4d*) were over expressed in peptide stimulated CD8*^+^* T cells while the protooncogene *c-Myc* was over expressed in virus stimulated T cells. The p18INK4c and p19INK4d gene products inhibit the function of CDK4 kinase, which in turn acts to phosphorylate Rb, relieving Rb-dependent inhibition of G_1_ to S progression [31]. The c-Myc gene product can act both directly and indirectly on CDK4 and thereby promote progression into S phase and DNA synthesis [32], [33].

**Figure 7:**
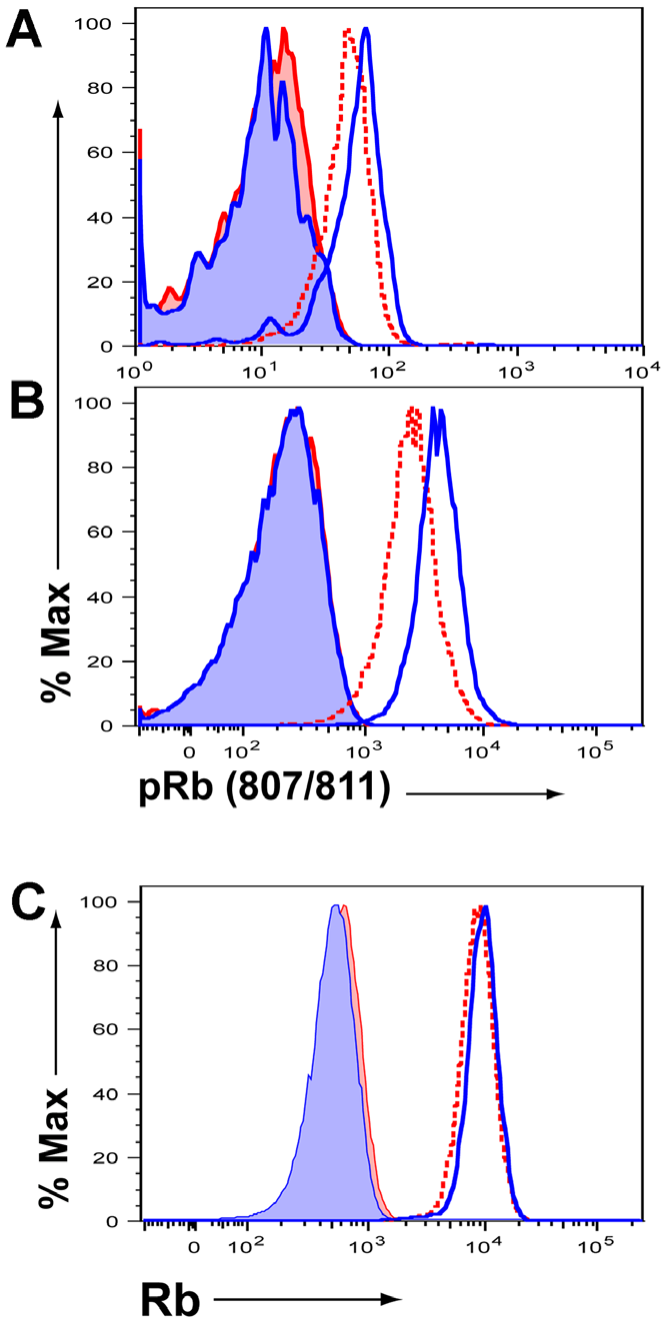
Retinoblastoma protein phosphorylation in proliferating CD8*^+^* T cells. A. Level of Rb phosphorylated at Ser_800/804_ in the CFSE labeled CL-4 T cells undergoing divisions 1 through 4 isolated from the DLN of influenza infected (78 hrs p.i., solid line –––) or VV-HA infected (54 hrs p.i., dotted line··········) animals. Isotype control antibody staining intensity for CL-4 T cells from influenza infected (shaded) and VV-HA Infected (stippled) donors are included. Data are representative of 3 exp. using 3 mice/exp. B. Analysis is as in Figure 7A except that CL-4 T cells from the lymph nodes of animals immunized i.v. with the synthetic PHA_533–541_ peptide epitope (36 hrs post-administration, dotted line··········) are compared to T cells from the DLN of influenza infected mice (78 hrs p.i., solid line –––) with a corresponding isotype control antibody staining for cells from virus infected (shaded) and peptide immunized (stippled) donors. Data are representative of 3 exp. using 3 mice/exp. C. Total Rb expression levels in CL-4 T cells analyzed in panel 7B with the corresponding isotype control antibody staining.

The above findings prompted us to ask whether there was a positive (or negative) correlation between the rapidity of cell division (and the duration of G_1_) and the expression of the retinoblastoma (Rb) protein. The transition of cells from G_1_ to the S phase of the cell cycle with the commitment to DNA synthesis is regulated by multiple factors [34], [35]. However, the Rb protein is believed to be a crucial controller of the transition from G_1_ into the S of the cell cycle which is regulated by this protein's phosphorylation state [16], [17], [36]. Site-specific phosphorylation of Rb (pRb) by late G_1_/early S cyclin dependent kinases at critical residues results in the dissociation of pRb from the transcription factor E2F allowing this transcription factor to initiate the expressions of genes required for the onset of DNA synthesis [16], [36]. We used a flow cytometry based approach to determine the level of Rb phosphorylation at Ser_800/804_ in proliferating CL-4 T cells undergoing divisions 1 through 4 in response to i.n. influenza A/PR/8 infection, VV-HA infection or immunization with the synthetic PHA_533–541_ peptide epitope. As Figure 7 demonstrates the CD8*^+^* T cells responding to influenza infection exhibited a higher level of pRb than T cells with a slower cell cycle time stimulated *in vivo* by VV-HA infection (Figure 7A) or synthetic peptide immunization (Figure 7B). These differences in pRb levels were not due to differences in the total expression level of Rb in the cells as the expression of Rb protein among the responding T cell populations were comparable (Figure 7C).

## Discussion

In this report, we have analyzed the *in vivo* proliferative response of CD8*^+^* T cells to several different antigenic stimuli including infection with the virulent viruses, type A Influenza and Vaccinia. We found that the T cells responding *in vivo* within the lymph nodes draining the site of virus infection in the respiratory tract are capable of dividing extremely rapidly, i.e., with initial cell cycle times of approximately 2 hrs at the start of T cell proliferation *in vivo*. As expected, this extremely short cell cycle time was reflected in the extremely large fraction of responding proliferating T cells in the S*+*G2/M phase of the cell cycle with most cells synthesizing DNA (as monitored by BrdU uptake). The cell cycle time of responding T cells *in vivo* was not fixed (uniform). We found that after undergoing > 5 divisions, the division rate of the activated T cells responding to infection began to slow. More importantly, the *in vivo* cell cycle time for initial T cell proliferation was also dictated by the type of antigenic stimulus with natural influenza infection triggering the most rapid cell division while *in vivo* immunization with a synthetic viral peptide epitope even when supplemented with strong adjuvants resulted in a markedly slower division rate, i.e., a cell cycle time of ∼6 hrs. We further found that the extremely rapid cell cycle time was characterized by a correspondingly markedly shortened G_1_ phase of the cell cycle and by elevated levels of the hyper-phosphorylated form of the tumor suppressor protein Rb.

Our finding of initial cell cycle times as short as 2 hrs for responding naïve murine T cells are considerably faster than the cell cycle times previously reported [25], [26], although in latter instances measurements were taken of the “average” over 24 hrs and as noted here after the initial burst of rapid cell division the division times for later cell divisions are slower. Our results are more compatible with recent evidence suggesting a rapid proliferative burst *in vivo* of the relatively small number of naïve antigen specific T cells in response to antigen [22]–[24].

Cell division in eukaryotes is a complex process controlled at multiple regulatory points throughout the cell cycle and is subject to both transcriptional and epigenetic regulation [34], [35]. We found that among these rapidly dividing T cells, approximately 90% of cells had taken up the thymidine analog BrdU of within 30 minutes of administration. Thus the majority of cells appeared to have transited through the G2/M and G_1_ phases of the cell cycle within 30 minutes. These findings reinforced the concept that the G_1_ phase of the cells cycle in these cells was dramatically shortened. Consistent with this concept we found that 85–95% of responding CD8*^+^* T cells with the 2 hr cell cycle times had > 2N DNA, i.e., were in S*+*G2/M. Rapid cell division with extremely short cell cycle times, like those reported here for activated murine CD8*^+^* T cells, have only previously been observed in embryonic tissue of rodents [37]. The requirement for extremely rapid DNA synthesis in cells dividing with short cell cycle times is achieved by initiating DNA synthesis at multiple origins of replication within the chromatin [38]. It is likely that along with the extremely short duration of G_1_ a similar mechanism operates in activated T cells undergoing extremely rapid initial proliferation.

We do not as yet understand the mechanism(s) regulating the tempo of cell division in these responding T cells but of note our gene expression array analysis implicates at least 3 potential target regulators of G_1_/S transition. Two of these targets, i.e., p18INK4c and p19INK4d which inhibit the activity of CDKs, are expressed at elevated levels in cells undergoing slow initial division times while the c-Myc proto-oncogene, which acts both directly and indirectly to stimulate the enzymatic activity of the CDKs regulating G_1_/S transition, is expressed at elevated levels in the CD8*^+^* T cells undergoing rapid division. The tumor suppressor Rb family proteins are critical regulators of the transition from G_1_ into S phase and the initiation of DNA synthesis [17], [39]. The association between rapid cell division and the level of phosphorylation of Rb suggests that in newly activated proliferating CD8^+^ T cells control of pRb may be critical for the control of cell cycle time and the duration of G_1._ The potential mechanistic link between the extent of phosphorylation of Rb and the rate of T cell division following antigenic stimulation is currently under active investigation in our laboratory.

An unexpected outcome of this analysis was the finding that the initial cell cycle time of CD8*^+^* T cells of responding *in vivo* was not fixed but rather dependent on the type of antigenic stimulus. Using this adoptive transfer model we have surveyed the impact of route and form of antigenic stimulus on the initial rate of T cell division. We should point out that division rates for CD8^+^ T cells responding to an antigenic stimulus in the draining lymph nodes were not restricted to either a 2 hr or a 6 hr initial cell cycle time. We found using this influenza T cell TCR tg model that initial cell cycle times of a short as 3–4 hrs were achievable with certain immunization regimens e.g., intravenous administration of influenza infected bone marrow dendritic cells (Figure 4C and Yoon and Braciale, unpublished data). However, immunization with synthetic peptide vaccines whether administered with adjuvants or as peptide pulsed dendritic cell populations uniformly resulted in T cell proliferative responses *in vivo* with longer initial division times while intranasal influenza infection triggers the fastest initial cell division rates *in vivo*. We cannot formally exclude the possibility that the differences in efficiency of stimulation leading to either a shorter or longer initial cell cycle time was a reflection of differences in the density of peptide/MHC complexes presented to the naïve CD8^+^ T cells in the draining lymph nodes. Thus, synthetic peptides and the recombinant vaccinia virus could produce a lower density of complexes resulting in a weaker initial stimulus for division and a slower initial cell cycle time in the responding T cells.

In connection with this we have also begun to evaluate the impact of TCR avidity on initial cell division time by analyzing the *in vivo* response of the CL-4 TCR tg T cells in the DLN to infection with a reverse genetics-derived mutant A/PR/8 strain containing two amino acid substitutions within the nine amino acid epitope within the transmembrane domain of the hemagglutinnin recognized by the CL-4 T-cells. This low avidity altered peptide ligand differs in avidity for the CL-4 TCR from the agonist A/PR/8 epitope by 2–3 orders of magnitude [40] and stimulates an initial cell division rate in the DLN which is one half that of the high avidity agonist epitope, i.e., 3–4 hrs/division (Kim and Braciale, unpublished observations).

We believe that the summation of signals received by the naïve T cell from TCR engagement of the peptide/MHC complex as well as from soluble and cell bound co-stimulatory ligands provided by APC likely programs the subsequent tempo of division of the responding lymphoblast. It will be of interest to determine whether this programming and signaling within the T cells via TCR and these costimulatory molecules can be linked to control of the expression of molecules such as p18INK4c, p19INK4d and c-Myc.

Proliferative expansion of T cells with a cell cycle time of ∼2 hrs could make the accumulating clonal progeny cells susceptible to mutation and chromosomal instability as there may not be adequate time for the rapidly cycling cells to undergo DNA repair [41]. The likely explanation for the selection and maintenance of this strategy by CD8*^+^* (and as our preliminary analysis suggests CD4*^+^*) T lymphocytes is the need to rapidly generate a large cohort of effector T cells directed to an invading pathogen from a very limited number of naïve pathogen-specific T cell precursors. Whether the slower initial division rate of CD8*^+^* T cells responding to Vaccinia virus infection reflects a potential mechanism which could be employed by microorganisms to limit the adaptive immune response by limiting the rate of initial proliferative expansion of the responding T cells remains to be determined. In regard to this it should be noted that the potential disadvantage of increased mutation frequency in activated T cells undergoing very rapid division is counterbalanced by the fact that the vast majority of these effector CD8*^+^* T cells will undergo apoptosis during the contraction phase of the immune response.

In conclusion, we have demonstrated that CD8*^+^* T cells responding to an antigenic stimulus can undergo extremely rapid proliferation with cell cycle times of as short as 2 hrs. This initial cell division time is not fixed but in part at least is dictated by the antigenic stimulus to which the naïve T cell responds. Since a rapid response of the adaptive immune system to infection is essential for clearance of most pathogens, understanding how cell cycle is regulated in T lymphocytes will not only yield important basic information on the biology of cell division but also may prove to be an important consideration in vaccine design.

## Materials and Methods

### Mice

The Clone 4 transgenic mice and Thy 1.1*^+^* BALB/c mice were provided by Dr. R. W. Dutton (Trudeau Institute, Saranac Lake, NY) and Dr. R. I. Enelow (Dartmouth College, Hanover, NH), respectively. DEMI-4 transgenic mice were constructed by us (manuscript in preparation) in collaboration with D. Moskephides (Medical College of Georgia/GA Research Inst.) and BALB/cANTac mice were purchased from Taconic Farms (Germantown, NY). All mice were maintained under pathogen free environment. Full details of the study are approved by the Institutional Biosafety Committee (#018-99) and the Animal Care and Use Committee (#2230) at the University of Virginia, VA.

### Virus and viral peptides

Mouse-adapted influenza virus A/PR/8/34 (H1N1) and A/JAPAN/305/57 (H2N2) were grown in allantoic cavity of 10 day chicken embryo (Charles River Laboratories, CT) as previously described [20]. Vaccinia virus expressing HA of A/PR/8 was a kind gift from Dr. J. W. Yewdell (NIH, Bethesda, MD). Synthetic peptide JHA529-537 (IYATVAGSL) and JHA210-219 (TYVSVGTSTL) of A/JAPAN and PHA533-541 of A/PR/8 (IYSTVASSL) were synthesized by University of Virginia Biomolecular Research Facility.

### Adoptive transfer of TCR tg CD8*^+^* T cells and virus infection

Naïve CD8*^+^* T cells were purified from the spleen by positive magnetic bead selection (MACS, Miltenyi Biotec) according to manufacturer's protocol. >97% of purified CD8*^+^* T cells were then washed with serum-free DMEM and labeled with 5 µM carboxyfluorescein diacetate succinimidyl ester in PBS (CFSE; Molecular Probes, Eugene, OR) for 10 min at room temperature. After extensive washing, typically 5×10^5^ labeled cells were transferred into recipient mice. Mice were rested at least for 24 hrs, then intranasally infected with 0.1 LD_50_ of influenza virus or 10^7^ pfu of VV-HA. When the mice were infected with intravenous injection, approximately 10^9^ EID_50_ unit of influenza virus was administered. The same dose of virus was used to infect CD11c*^+^* DC *in vitro.* When the antigenic peptides were used to stimulate *in vivo*, 100∼250 µl of 1 µM peptides in endotoxin free PBS were injected i.v. with or without TLR ligand agonists individually or together. Poly I:C (TLR-3) and R848 (TLR-7) were purchased from Sigma and CpG ODN 1668 was produced by IDT (Coralville, IA). All TLR ligand agonists were used at 100 µg per mouse.

### Blocking entry of T cells into lymph nodes by MEL-14 antibodies

To block further entry of T cells into lymph nodes, 100 µg of intact IgG2a monoclonal anti CD62L (MEL-14) was injected i.v. into the mice 24 hrs p.i. as described previously [21] with influenza virus infection.

### *In vitro* culture

2×10^6^ of purified and CFSE-labeled transgenic CD8*^+^* cells were incubated with 2×10^7^ of peptide-loaded BALB/c splenocytes in complete IMDM (Iscove's modified Dulbecco's media supplemented with 10% FBS, 10 U/ml penicillin G, 10 µg/ml streptomycin sulfate, 2 mM L-glutamin, and 0.05% 2-ME without IL-2). Splenocytes were pulsed with 100 nM peptides for 1 hr at RT. Free unbound peptides were washed away before adding the splenocytes to the co-culture.

### Measurement of DNA content

The prepared and fixed cells were washed with PBS and incubated with 1∶7 diluted DRAQ-5 (Biostatus, UK) at 37°C for 15 min. The cells were immediately analyzed using a BD FACS Calibur or Canto within 1 hr. The doublets were discriminated and the gates for G_1_ and S*+*G_2_/M were determined according to the DNA profile of undivided cells in the same sample tube.

### Detection of BrdU

1 mg of BrdU (Sigma) in 50 µl sterile PBS was administrated i.v. into the mice. The single cell lymph node suspensions were prepared immediately after excision of nodes from each mouse and kept in FACS Buffer with saturating amount of thymidine (20 mg/ml) on ice to block further incorporation of BrdU during excision of other samples. Anti-BrdU staining for flow cytometer was performed with BrdU Flow Kit (BD Biosciences) according to their protocol.

### Preparation of tissue lymphocytes and flow cytometry

At the time of collection, the lymph nodes, spleens, or lungs were removed and placed in ice cold FACS staining buffer (PBS with 2% FBS and 0.02% NaN3), then subsequently disrupted and passed through a cell strainer (70 mm; BD Falcon, Bedford, MA). For enzymatic liberation of cells, the disrupted tissues were incubated with collagenase type XI (125 U/ml), hyaluronidase type I-s (60 U/ml), and DNase I (60 U/ml)[Sigma] in Mg and Ca free HBSS (Gibco) at 37°C for 30 min. 10^6^ cells were incubated with anti-CD16/32 (2.4G2) to block non-specific FcR binding, stained with anti-CD8α (53-6.7), anti-Thy-1.2 (53-2.1), anti-CD69 (H1.2F3), and anti-CD25 (PC61)[BD Pharmingen], and fixed in FACS lysing solution (BD Bioscience). For intracellular staining, the cells were fixed and permeabilized with 90% MeOH. Anti-Rb (G3-245, BD) antibody was conjugated to the Alexa Fluor by mAb labeling kits according to the supplier's (Molecular Probes) protocol. Anti-human pRb (807/811) (J112-903) was purchased from BD. Flow cytometric acquisitions were done with FACSCalibur or FACSCanto (BD Pharmingen) and analyzed with FlowJo software (TreeStar).

### Enrichment of CD11c*^+^* cells using Flt3L

The original pUMVC3-hFlex (Flt3 ligand [Flt3L]) plasmid was a kind gift from Hardy Kornfeld (University of Massachusetts Medical School, Worcester, MA). The enrichment of CD11c*^+^* DC in mice was done according to the previous protocols reported [27]. Briefly, 2 ml of endotoxin-free Flt3L plasmid (5 µg/ml) was injected intravenously rapidly within 10 seconds into the mice. The plasmid was injected one more time at day 6 after first injection, then the spleens were collected after another 6 days after second injection. The splenic CD11c*^+^* DC were purified using CD11c magnetic beads by suppliers protocol (Miltenyi). For maturation of CD11c*^+^* DC *in vitro,* cells were incubated for 24 hrs in complete IMDM with LPS from *Salmonella abortus equi* (1 µg/ml) (Sigma).

### Virus infection of dendritic cells

High concentration of viruses (approximately 10^9^ EID_50_ unit of influenza virus or 5×10^8^ pfu of VV-HA) was incubated with 10^6^ dendritic cells in 500 µl of serum-free PBS for 10 min in ice and 30 min at 37°C. Then, the cells were resuspended in endotoxin-free PBS for injection after extensive washing to remove unbound virus particles.

### Affymetrix gene chip analysis

Thy1.1*^+^* mice adoptively transferred with CFSE labeled 1×10^6^ of thy1.2*^+^* CL-4 CD8*^+^* T cells were infected intranasaly with influenza virus A/PR/8 or injected intravenously with peptide PHA533-541. The lymphocytes from pooled LNs were subjected to thy1.2 positive selection by magnetic bead selection at the time indicated post infection. The cells which had undergone 1 through 5 divisions according to CFSE dilution, were identified and collected using a Becton Dickinson FACSVantage SE Turbo Sorter (Flow Cytometry Core Facility at Univ. of VA) and RNAs were purified using RNAeasy kit (Qiagen, Valencia, CA) according to manufacturer's protocol. The RNAs were used for Affymetrix GeneChip analysis on Mouse Genome 430 2.0 Array (Biomolecular Research Facility at Univ. of VA). The gene array data were analyzed by Agilent Bioanalyzer and intensity files obtained by Affymetrix MAS program were normalized by dChip. Heatmap profiles for genes of interest were produced by Excel program.

## Supporting Information

Figure S1 Effect of method of cell liberation from DLN, T cells isolated from sites outside the DLN, and dose of transferred T cells on the CD8^+^ T cell proliferation in the DLN. (DOC)

Figure S2 DNA synthesis by CL-4 tg CD8^+^ T cells in the NDLN. (DOC)

Table S1 Summary of the percentage of cells in S+G2/M (>2N DNA content) among the responding CD8^+^ T cells in the DLN at 78 hrs p.i. (DOC)
