# Supplementary material for: The Cell Cycle Time of CD8+ T Cells Responding In Vivo Is Controlled by the Type of Antigenic Stimulus
Source: PLoS One. 2010 Nov 8;5(11):e15423. doi: 10.1371/journal.pone.0015423 (PMC2975678; doi:10.1371/journal.pone.0015423)
Supplement: Figure S1 — Effect of method of cell liberation from DLN, T cells isolated from sites outside the DLN, and dose of transferred T cells on the CD8+ T cell proliferation in the DLN. (DOC) [file pone.0015423.s001.doc]

**Figure S1. Effect of method of cell liberation from DLN, T cells isolated from sites outside the DLN, and dose of transferred T cells on the CD8^+^ T cell proliferation in the DLN.**

**(A)**

**
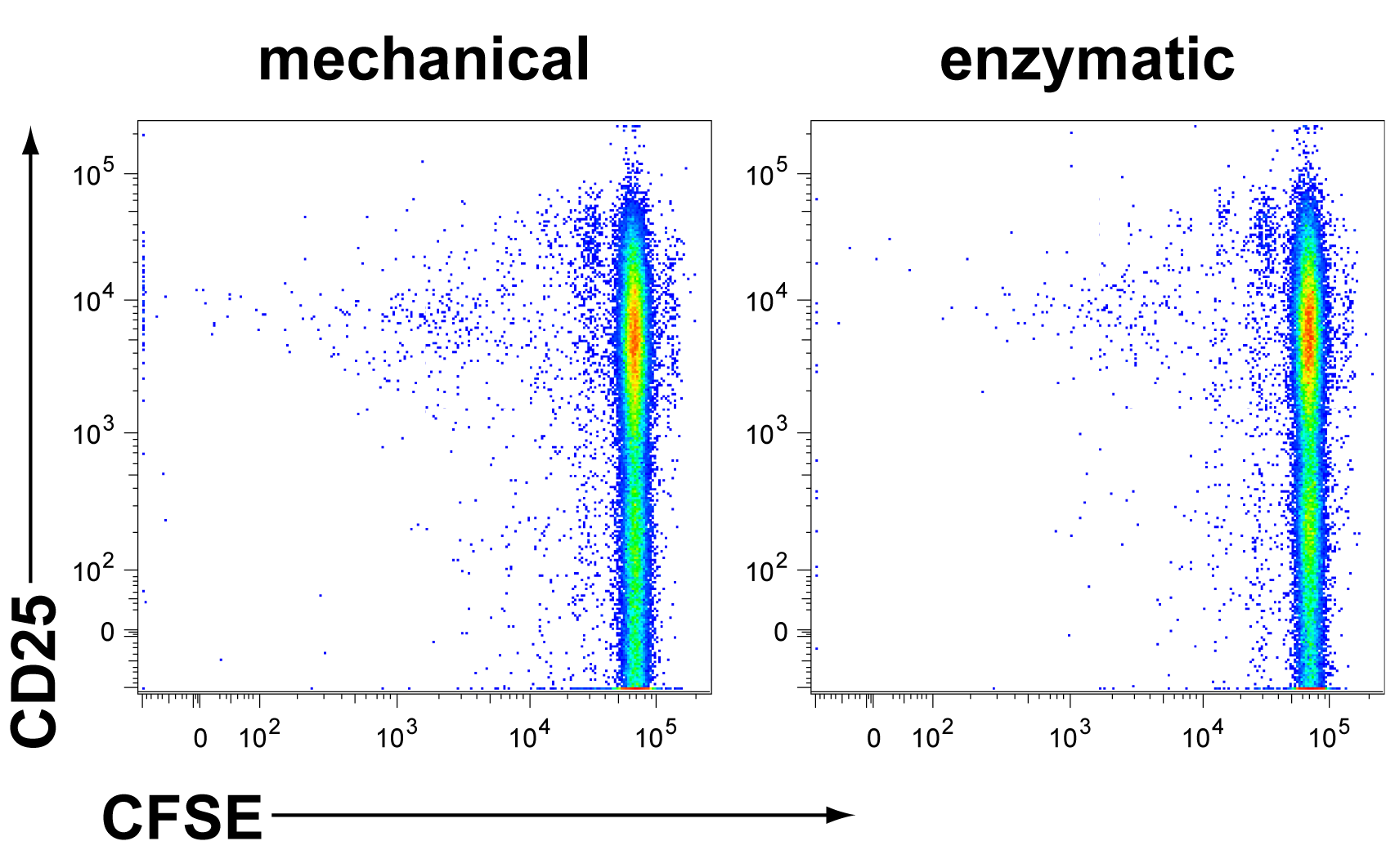
**

**(B)**

**
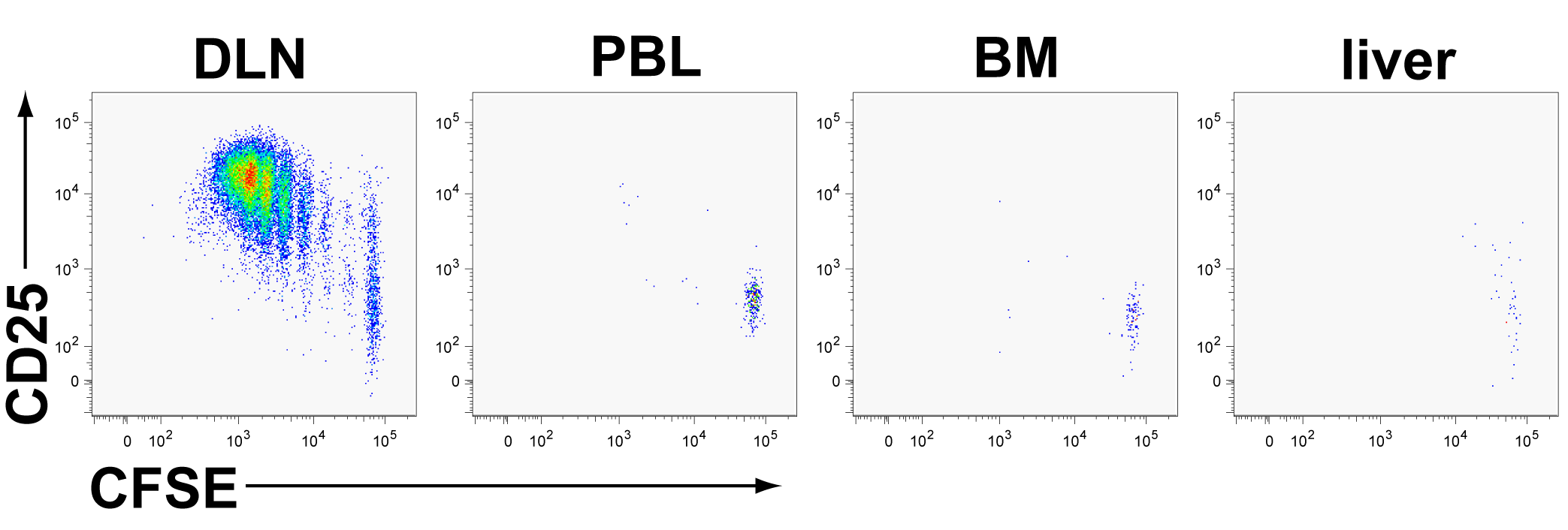
**

**(C)**

**
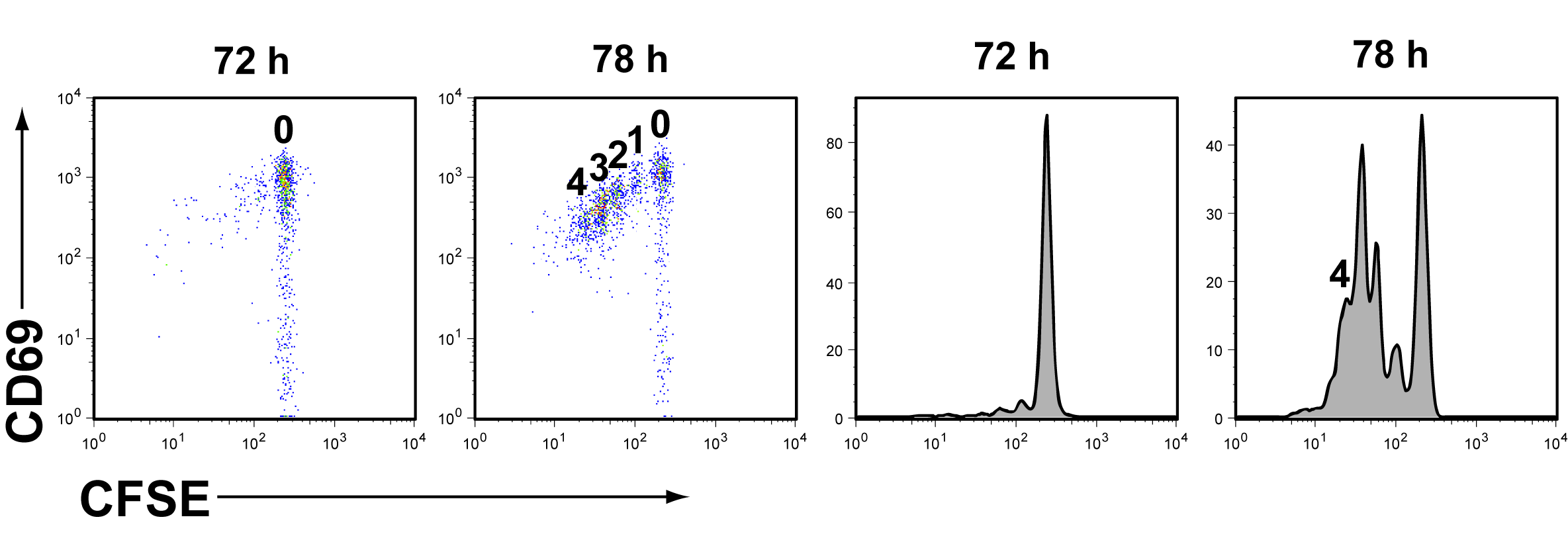
**

**(A)** The DLN from recipients of CFSE label CL-4 T cells were harvested at 72 hrs p.i. with influenza A/PR8 and subjected to mechanical or enzymatic digestion (Materials and Methods) followed by flow based analysis of CFSE intensity of the liberated cells. Values are representative of two exps. using 2 mice/exp.

**(B)** Intensity of CFSE staining of dye labeled CL-4 T cells responding in the lung draining lymph nodes (DLN) Peripheral blood (PBL), bone marrow (BM) , and liver 4 days after influenza infection. As previously reported (Lawrence and Braciale, 2004) proliferating CL-4 T cells were not detected in the non draining lymph nodes or spleen of infected animals at this time ( data not shown). Pooled data from 3 mice in one exp.

**(C)** *In vivo* proliferative response of 5X10^4^ transferred CL-4 T cells to influenza infection has determined by CFSE dye intensity of T cells liberated from the excised DLN at the indicated times p.i. Pooled cells from the DLN of 10 mice in one exp.
