# Supplementary material for: The Cell Cycle Time of CD8+ T Cells Responding In Vivo Is Controlled by the Type of Antigenic Stimulus
Source: PLoS One. 2010 Nov 8;5(11):e15423. doi: 10.1371/journal.pone.0015423 (PMC2975678; doi:10.1371/journal.pone.0015423)
Supplement: Figure S2 — DNA synthesis by CL-4 tg CD8+ T cells in the NDLN. (DOC) [file pone.0015423.s002.doc]

**Figure S2. DNA synthesis by CL-4 tg CD8*^+^* T cells in the NDLN.**


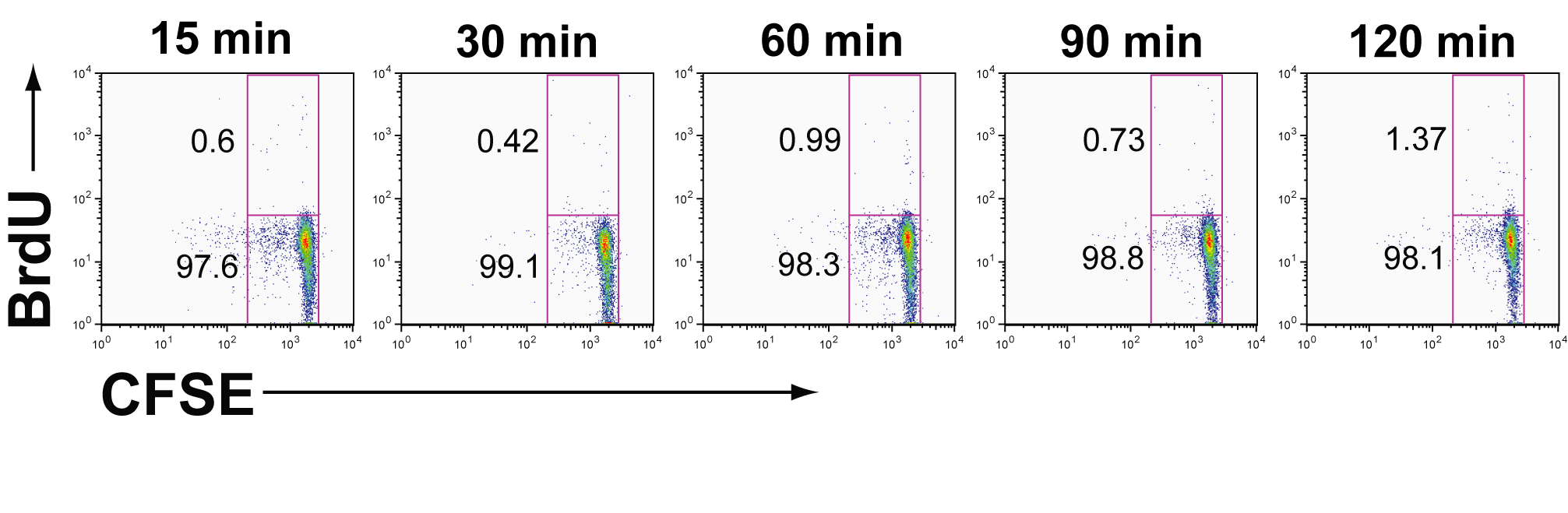


Uptake of BrdU *in vivo* by CFSE labeled CL-4 T cells isolated from non draining lymph nodes at day 3.5 p.i. following BrdU administration for the indicated time period. Pooled data from 3 mice in one experiment.
