# Supplementary material for: The Cell Cycle Time of CD8+ T Cells Responding In Vivo Is Controlled by the Type of Antigenic Stimulus
Source: PLoS One. 2010 Nov 8;5(11):e15423. doi: 10.1371/journal.pone.0015423 (PMC2975678; doi:10.1371/journal.pone.0015423)
Supplement: Table S1 — Summary of the percentage of cells in S+G2/M (>2N DNA content) among the responding CD8+ T cells in the DLN at 78 hrs p.i. (DOC) [file pone.0015423.s003.doc]

**Supplemental Table**

**Table S1.** **Summary of the percentage of cells in S*+*G2/M (>2N DNA content) among the responding CD8*^+^* T cells in the DLN at 78 hrs p.i.**

|  | | | | | | |
| --- | --- | --- | --- | --- | --- | --- |
|  | **% of CL-4 T cells in S+G_2_/M** | | | | | |
|  | Division Number | | | | | |
|  | 0 | 1 | 2 | 3 | 4 | 5 |
| 78h | 22.8 ± 2.77 | 80.4 ± 4.42 | 93 ± 1.47 | 94.8 ± 0.9 | 91.4 ± 0.48 | 73.3 ± 4.86 |
|  | | | | | | |

Percentage of CL-4 TCR transgenic T cells in S*+*G2/M by DNA content in the DLN of influenza A/PR8-infected mice at 78 hrs p.i. Percentages are for the indicated cell division number as defined by CFSE dye intensity. Values are the Mean ± SEM of 6 independent experiments with 1-3 animals per experiment.
